# Automated quality assurance of imaging dose and protocol adherence in computed tomography radiotherapy planning using TotalSegmentator-based segmentation

**DOI:** 10.1007/s00066-025-02494-w

**Published:** 2025-11-28

**Authors:** Niklas A. Lackner, Andre Karius, Tobias Brandt, Oliver J. Ott, Florian Putz, Vratislav Strnad, Matthias S. May, Rainer Fietkau, Christoph Bert, Juliane Szkitsak

**Affiliations:** 1 Department of Radiation Oncology, Universitätsklinikum Erlangen, Friedrich-Alexander-Universität Erlangen-Nürnberg (FAU), Universitätsstr. 27, 91054 Erlangen, Germany; 2 Comprehensive Cancer Center Erlangen-EMN (CCC ER-EMN), Erlangen, Germany; 3 Comprehensive Cancer Center Alliance WERA (CCC WERA), Erlangen, Germany; 4 Bavarian Cancer Center Erlangen (BZKE), Erlangen, Germany; 5 Institute of Radiology, Universitätsklinikum Erlangen, Friedrich-Alexander-University (FAU), Erlangen, Germany

**Keywords:** Workflow optimization, Computed-tomography dose optimization, Retrospective analysis, Deep learning, Radiation exposure

## Abstract

**Purpose:**

Computed tomography (CT) scans are vital for radiotherapy planning, providing essential data for dose calculations. This study retrospectively evaluated imaging doses, scan lengths, and protocol adherence to support imaging optimization and reduce patient radiation exposure.

**Methods:**

CT data from patients undergoing external beam radiotherapy and brachytherapy in the period 04/2021 to 12/2024 were retrieved from the institutional picture archiving and communication system (PACS). Imaging doses (volumetric CT dose index [CTDIvol] and dose length product [DLP]) were extracted from dose reports. Automated organ segmentation was used to assess standard operating procedures (SOPs) adherence by estimating anatomical scan length differences. Additional quality assurance checks assessed protocol and imaging consistency.

**Results:**

Brain protocols exhibited the highest CTDIvol (73 ± 12 mGy), while head and neck protocols had higher DLP values (3212 ± 757 mGy·cm). The lung 4D protocol showed a higher effective dose (23 ± 9 mSv) compared to the standard lung protocol. Notable anatomical scan length differences were observed at the lower boundary in the upper abdomen (120 ± 75 mm) and spine (155 ± 159 mm), indicating opportunities for workflow improvement.

**Conclusion:**

Enhancing CT workflows for radiotherapy patients is important and feasible. Dose and scan length analyses suggest that revising institutional SOPs, optimizing X‑ray tube modulation, and refining scan length boundaries should be considered to achieve this goal.

**Supplementary Information:**

The online version of this article (10.1007/s00066-025-02494-w) contains supplementary material, which is available to authorized users.

## Introduction

Computed tomography (CT) is essential for radiotherapy planning [1], enabling precise localization of targets and organs-at-risk [2] while also providing electron density data for dose calculation [3, 4].

A constant tube voltage is typically used in radiotherapy CT to ensure consistent HU-to-electron density conversion and reduce dose calculation errors [5, 6]. In contrast, diagnostic CT often varies tube voltage to optimize dose. Techniques like DirectDensity (Siemens Healthineers AG, Forchheim, Germany) may reduce dependence on calibration curves, enabling flexible electron density mapping [7].

Over the past two decades, radiotherapy CT protocols have evolved alongside increasing scanner complexity, incorporating features like automatic exposure control and iterative reconstruction to improve image quality while reducing patient dose [8, 9]. Dose descriptors such as volumetric CT dose index (CTDI_vol_), dose length product (DLP), and effective dose (E) support this balance between image quality and exposure [10–13], especially in light of these technological advancements [14–16].

While diagnostic CT is subject to legal reference levels in many countries [17–19], radiotherapy CT is less regulated. Recent guidelines, such as from the German Society for Radiation Oncology (DEGRO), propose nonbinding recommendations for scan protocols and dose management [20]. Previous efforts have highlighted protocol variation and proposed reference dose levels for radiotherapy CT based on data from 68 scanners [21, 22]. However, international consensus remains limited.

Given the basic role of CT in radiotherapy, early quality assurance (QA) is critical [23, 24]. Metal artifacts, contrast agents, limited field-of-view (FOV), or poor patient positioning can affect the entire workflow. Errors at this stage may propagate through planning and treatment, underscoring the value of robust CT QA [25–28].

This retrospective study evaluated imaging dose metrics, anatomical coverage, and protocol adherence using automated segmentation, with additional evaluations addressing imaging consistency to support standardization and improve patient safety.

## Materials and methods

### CT scanning and protocols

CT data were acquired using a SOMATOM go.Open Pro scanner (Siemens Healthineers AG, Forchheim, Germany), specifically designed for radiotherapy applications. All CT scans in this study were performed exclusively as planning CTs for treatment simulation. For all scans, tube current modulation (TCM) via the Siemens CARE Dose 4D algorithm was activated, a constant tube voltage of 120 kVp was set, and the scanner configuration was adjusted for adult body size. The TCM dynamically adjusts the tube current based on organ-specific characteristics, using angular and longitudinal modulation [29, 30]. Spiral scanning was used in all protocols except for the 4DCT protocol, which utilized axial scanning in the Direct i4D scan mode [31–35]. The scanner features extended field-of-view reconstruction (HDFoV) for cases requiring an expanded field of view [36]. The organ characteristics varied depending on the protocol and are provided in Table 1. Scans for stereotactic treatment (STX) use a slice thickness of 1 mm, while for stereotactic radiosurgery (SRS), we use 0.6 mm. For all other scans, a standard slice thickness of 3 mm is applied. During the routine retrospective QA process and preparation of this work, it was identified that the head & neck and spine CT protocols delivered considerably higher doses compared to achievable levels reported by Wood et al. [22]. In early 2024, the TCM reference characteristics were therefore updated, changing from ‘head’ to ‘neck’ (head & neck) and ‘abdomen’ (spine) profiles for the respective protocols. This adjustment aligned the modulation behaviour more closely with the anatomical attenuation patterns of the scanned regions, improving dose efficiency while maintaining image quality.

**Table 1:** Summary of scanning protocols, including body regions, groups, and TCM-related parameters (organ characteristics). It is noteworthy that the organ characteristics were adjusted in two EBRT scan protocols during the study: head & neck and spine, both transitioning from head-specific to neck-specific and abdomen-specific protocols. Additionally, a summary of anatomical measurements and corresponding FOV specifications according to internal standard operating procedures for clinical imaging protocols, including reference values for missing organ lengths based on assessments from the vertebral body lookup table

Body region groups	Body region	Organ characteristic	Anatomical FOV according to internal SOPs	Reference for missing lengths
Brain	Brain	Head	*Cranial:* cranial start of skull +1 cm cranial	*Cranial:* Skull
			*Caudal*: end of C4	*Caudal:* C4, C3, C2, …
H&N	H&N (EBRT & Brachy)	Head (23) Neck (24)	*Cranial:* cranial start of skull +1 cm cranial	*Cranial:* Skull
			*Caudal:* caudal end of lung	*Caudal:* T12, T11, T10, …
Thorax	Lung	Thorax	*Cranial:* mastoid (equivalent organ: cranial end of C1)	*Cranial:* C1, C2, C3, …
	Breast (EBRT & Brachy)		*Caudal:* caudal end of liver	*Caudal*: L2, L1, T12, …
4D	Lung Upper abdomen	Respiratory	*Cranial:* cranial end of lung	*Cranial*: C7, C8, C9, …
			*Caudal:* caudal end of lung	*Caudal:* T12, T11, T10, …
Upper abdomen	Liver Kidney Pancreas	Abdomen	*Cranial*: tracheal bifurcation (equivalent organ: cranial end of T4)	*Cranial:* T4, T5, T6, …
			*Caudal:* iliac crest (equivalent organ: cranial end of hip)	*Caudal:* L3, L2, L1, …
Abdomen	Paraaortic Bladder	Abdomen	*Cranial:* 3 cm cranial diaphragm (equivalent organ: cranial end of liver)	*Cranial:* T10, T11, T12, …
			*Caudal:* trochanter minor (equivalent organ: caudal end of hip)	*Caudal:* L5, L4, L3, …
Pelvis	Anal Groin Pelvis Hip Prostate (EBRT & Brachy) Rectum Cervix (EBRT & Brachy)	Abdomen	*Cranial:* diaphragm (equivalent organ: cranial end of liver)	*Cranial:* T10, T11, T12
			*Caudal:* 10 cm caudal trochanter minor (equivalent organ: 10 cm caudal end of hip)	*Caudal:* L5, L4, L3
Spine	Spine	Head (23)	*Cranial:* mastoid (equivalent organ: cranial end of C1)	*Cranial:* C1, C2, C3, …
		Abdomen (24)	*Caudal:* iliac crest (equivalent organ: cranial end of hip)	*Caudal:* L3, L2, L1, …

Note: Entities not explicitly listed have been evaluated under the closest related protocols

*H&N* head & neck; *4D* four-dimensional; *EBRT* external beam radiation therapy; *SOPs* standard operating procedures, *TCM* tube current modulation, *FOV* field of view, *Brachy* brachytherapy

### Data retrieval and extraction

Data retrieval and extraction were conducted using the DCM4CHE toolkit [37] to query the institutional picture archiving and communication system (PACS), where an automated bash script filtered and retrieved imaging studies from April 2021 to December 2024. Each study included the structured reports (dose and patient report) and the accompanied imaging series. Protocols with a total occurrence of fewer than 25 cases such as emergency cases or other specific cases were excluded from the analysis. This process was cross-checked against data from Mosaiq (Elekta AB, Stockholm, Sweden), an oncology information system used to manage patient treatment data, to ensure completeness. A MATLAB (version R2019b, The MathWorks Inc., Natick MA, USA) script was developed to extract DICOM metadata from structured report files, inspired by previous work from Dave and Gingold [38]. The accuracy of the algorithm was tested through manual verification on a subset of the dose report images and their corresponding DICOM metadata. A comprehensive overview of all included external beam radiation therapy (EBRT) and brachytherapy datasets, including the respective dose descriptors, is provided in the supplementary information S1, Table A1. All patient data used in this study were included as part of clinical routine practice and analyzed retrospectively. All procedures were conducted in accordance with the ethical standards of the institutional research committee and with the Declaration of Helsinki from 1964 and its subsequent amendments.

### Data analysis

#### Imaging dose metrics

Imaging doses were extracted from the dose reports, which include individual imaging events such as topograms for defining the FOV and subsequent volumetric scans. For each volumetric scan, parameters such as CTDI_vol_, scan lengths, DLP, and effective dose were evaluated. CTDI_vol_ values, as reported in the dose reports, are based on standardized phantom diameters of 16 and 32 cm, which reflect typical clinical phantom sizes used for dose estimations. Effective dose was estimated by applying AAPM-defined k‑factors for each body region [12]. These k‑factors account for variations in radiation sensitivity across different body regions and are based on standardized models for the calculation of E. For each scan, the DLP was multiplied by the k‑factor corresponding to the anatomical region covered to estimate effective dose. The values of effective dose derived from this method show strong agreement with those obtained through more rigorous calculation approaches, with deviations from the mean typically within 10–15% [39]. Table A1 in the supplementary information provides details on the specific k‑factors applied for various body sites for clarity and cross-referencing. The imaging dose analysis was compared to proposed values from Wood et al. [22] (for details see supplementary information S1 Table A2).

To provide context for the QA framework, CTDI_vol_, DLP, and effective dose were evaluated jointly. CTDI_vol_ reflects scanner-reported output normalized to phantom size and is useful for monitoring protocol consistency. DLP incorporates the actual scan length and therefore provides a more patient-specific measure of imaging exposure. Effective dose, derived from DLP using region-specific k‑factors, represents a risk-related quantity that facilitates comparison across body regions and protocols. Considering all three descriptors allows for both technical QA of acquisition parameters and clinical interpretation in terms of patient exposure.

#### Anatomical landmark check

**Fig. 1:**
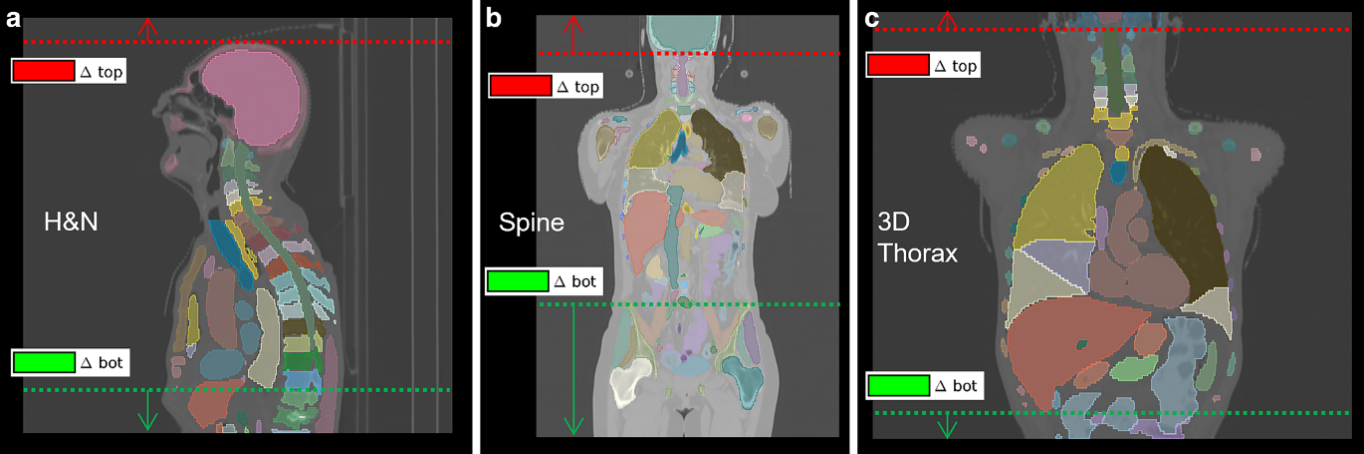
Schematic of anatomical field-of-view (FOV) assessment and standard operating procedure (SOP)-defined boundaries. Dashed lines indicate expected FOV per SOPs; red and green dashed lines denote cranial (∆top) and caudal (∆bot) deviations, respectively. Upward (red) and downward (green) arrows indicate the positive direction of ∆top and ∆bot, corresponding to cranial and caudal overcoverage. Example cases include head & neck (left), spine (center), and 3D thorax (right). Segmentations were overlaid in 3D Slicer [41]. ∆top and ∆bot values represent anatomical scan length deviations from SOP targets (positive = overcoverage; negative = undercoverage). Anatomical FOV and SOP specifications are listed in Table 1

The TotalSegmentator deep learning-based tool, introduced by Wasserthal et al. [40], was employed to segment specific organs within the scans, and for creating detailed anatomical landmarks. These landmarks were then used to assess adherence to SOPs for CT imaging, ensuring that scans align with protocol-defined anatomical coverage. Anatomical scan length differences were calculated to measure coverage variation from SOP targets, where ∆top represents the difference at the cranial border and ∆bot represents the difference at the caudal border of the scan (Fig. 1). Brachytherapy scans were excluded from the comparison because their field of view is often determined on an ad hoc basis, depending on individual clinical needs. Similarly, scans for protocols such as the axilla or extremities were excluded because they also often require ad hoc decisions driven by patient-specific needs and exhibit high variability in clinical implementation, making consistent comparisons challenging.

To estimate negative scan length differences, we developed a standardized approach using a vertebral body lookup table (VB-LUT), which provides predefined anatomical measurements for specific regions when scans are shortened or truncated at anatomical borders. The VB-LUT includes reference lengths for the skull and vertebral bodies: 210 mm for the skull, 25 mm for C1–C2, 20 mm for C3–C7, 25 mm for T1–T12 and 30 mm for L1–L5 (more details in supplementary information S2).

The method of estimating positive scan length differences was reliable within one slice thickness (typically 3 mm) for positive values, which we manually validated in a sample of scans. In contrast, estimating negative differences was less reliable due to challenges in accurately estimating scan differences in truncated scans based on general anatomical assumptions, which do not account for individual variations in human anatomy. While this method was reliable for smaller truncations, its accuracy decreased notably when larger portions of the scan were missing. Deviations were particularly pronounced if the patient’s anatomy substantially differed from the reference values, such as in exceptionally tall or short individuals.

In cases where direct segmentation of structures was not available, or essential structures were cut at the borders, we utilize equivalent height-level organs to approximate missing sections. For example, the cranial end of C1 can serve as a reference point for estimating the cranial border, such as the ‘mastoid’ in thoracic scans. Detailed specifications, including cranial and caudal reference points and the corresponding anatomical FOV from SOPs, are summarized in Table 1.

#### Additional QA checks

We implemented a series of QA checks to evaluate image acquisitions for their adherence to clinical standards. Contrast agent application volumes were extracted and assessed from the structured report patient protocol. Iterative metal artifact reduction (iMAR, Siemens Healthineers AG, Forchheim, Germany) checks were performed using image data and HU thresholding (HU > 4000) to identify metal. Topogram lengths were compared against volumetric scan lengths to determine if topogram lengths were occasionally shorter than the volumetric scans. An image slice count below 399 was confirmed to comply with institutional protocol limits, as one of the image guidance systems used on the treatment accelerator rejects scans exceeding this threshold. Segmented organs were used to verify the breathing phase for thoracic scans: if a minimum of two scans in different breathing modes were available (e.g. expiration and free breathing), lung volumes were compared for plausibility. Bladder filling assessments, based on volumetric evaluations and classification into filling states (empty: < 200 mL, partially filled: 200–400 mL, full: > 400 mL), were performed for patients undergoing bladder, rectal, prostate, anal, and cervical imaging. This follows the clinical recommendation to standardize protocols for bladder filling to spare small pelvic bowel loops [20]. We also evaluated the misapplication of the thorax protocol for abdomen imaging by analyzing scan length differences. Scans were flagged as misapplied when the cranial end extended more than 10 cm below and the caudal end more than 10 cm above the intended thorax coverage. Finally, we conducted systematic FOV checks for axilla protocols to ensure sufficient anatomical coverage. Specifically, we assessed whether the upper half-circle edge of the reconstructed image contained non-air values, indicating potential truncation of the patient’s skin or anatomy. Additionally, we examined a predefined check position outside the standard reconstruction field, where pixel values would only be present if an HDFoV reconstruction was applied. If truncation was detected, we manually reviewed the images to verify the findings. Axilla protocols were particularly suited for this check, as ensuring full coverage of the relevant anatomy is considered clinically important. A detailed description of the automated data-retrieval, segmentation, and QA workflow, including algorithmic logic and decision criteria, is provided in the supplementary information S3.

## Results

### Imaging dose metrics

**Fig. 2:**
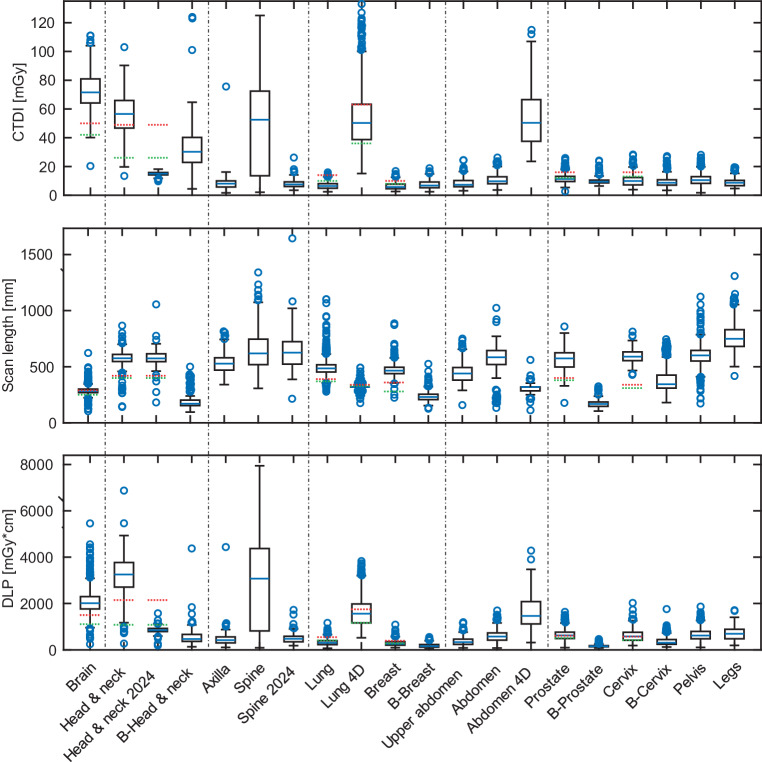
Volumetric computed tomography (CT) dose index (CTDI_vol_), scan length, and dose length product (DLP) for various scan protocols compared to proposed (red) and achievable (green) values from Wood et al. [22]. Spine protocols show greater variability, indicated by wider interquartile ranges. Body region groupings (dashed lines) aid comparison across anatomical areas. Brachytherapy protocols are prefixed with “B” (e.g. “B Breast”)

**Fig. 3:**
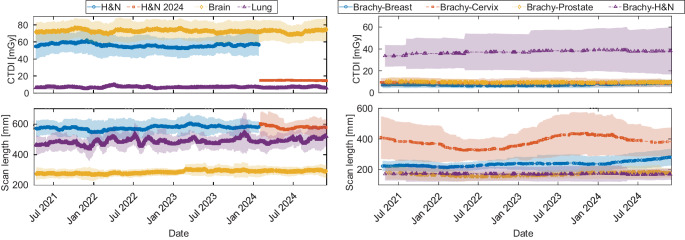
Temporal trends in volumetric computed tomography (CT) dose index (CTDI_vol_ ) and scan length using a 12-week moving average. Lines represent moving averages; shaded areas indicate standard deviations. Left: External beam radiation therapy (EBRT) scans (head & neck [H&N], brain, lung). The blue and red curves correspond to head & neck before and after the 2024 protocol adjustment (“organ characteristic” change). The reduced red standard deviation in CTDI_vol_ reflects lower dose variability due to the more homogeneous modulation setting. Right: Brachytherapy (brachy) scans (breast, cervix, prostate, head & neck). Greater variability in brachytherapy protocols reflects their individualized imaging approaches and smaller case numbers. For the initial time period, the 12-week moving average was computed using all available data within the fixed ±6-week window around each date, which may result in wider uncertainty at the beginning of each plot

Imaging doses from a total of 10,107 dose reports were retrieved from the institutional PACS. We evaluated radiation exposure across various CT protocols, comparing observed CTDI_vol_, scan length, DLP and estimated E. Notably, the brain protocols exhibited the highest CTDI_vol_ of 73 ± 12 mGy, while the head & neck protocols, despite a lower mean CTDI_vol_ of 56 ± 12 mGy, recorded a higher DLP at 3212 ± 757 mGy·cm due to its longer mean scan length of 576 ± 58 mm. Following the organ characteristic adjustment in 2024, the mean DLP for head & neck protocols dropped to 856 ± 121 mGy·cm. Among thoracic protocols, the 4D lung protocol had a higher effective dose of 23 ± 9 mSv compared to the standard lung protocols (4 ± 2 mSv), reflecting increased dose requirements for motion-resolved imaging. In abdominal imaging, the upper abdomen protocol demonstrated a moderate effective dose of 6 ± 3 mSv, with a mean scan length of 444 ± 82. Spine protocols were among the highest in terms of CTDI_vol_, averaging 60 ± 27 mGy, with an effective dose of 11 ± 5 mSv, and showed a broad scan length distribution with a mean of 644 ± 168 mm. The organ characteristic adjustment in 2024 specifically reduced the CTDI_vol_ for spine protocols to 8 ± 3 mGy. Thoracic, abdominal, and pelvic protocols, excluding 4D protocols, remained below a mean CTDI_vol_ of 15 mGy. Among them, thoracic and upper abdomen protocols showed similar values and were the lowest, while abdomen and pelvic protocols exhibited slightly higher but comparable values. S1 Table A1 in the supplementary information summarizes these dose metrics across EBRT and brachytherapy protocols. Figure 2 illustrates the distributions of CTDI_vol_, scan length, and DLP across various CT protocols. The green and red dashed lines represent the proposed and achievable reference values, respectively, from the UK study by Wood et al. [22] for radiotherapy treatment planning CTs, serving as a benchmark for comparison. The 12-week moving average of the CTDI_vol_ and scan lengths are presented in Fig. 3, where lines represent the moving average, and error bands indicate the running standard deviation. For EBRT protocols, brain, lung, and head & neck (H&N) scans show stable scan lengths. In brachytherapy protocols, scan lengths are generally stable, except for cervix protocols, which exhibits greater variability. This variability may be influenced by the smaller sample size in brachytherapy protocols. Regarding CTDI_vol_, most EBRT and brachytherapy protocols remain stable over time. However, H&N EBRT scans show a noticeable decrease in CTDI_vol_ in 2024, due to the organ characteristic adjustment.

### Anatomical landmark check

For ∆top, brain (11 ± 17 mm), head & neck (9 ± 32 mm), thorax 4D (17 ± 29 mm), and abdomen (16 ± 65 mm) exhibited the smallest deviations, while pelvis (−15 ± 57 mm) showed slightly larger but still moderate variations. More pronounced undercoverage was observed for thorax 3D (−45 ± 48 mm) and upper abdomen (−40 ± 51 mm), with the most substantial deviations seen in spine (−68 ± 163 mm). For ∆bot, brain (24 ± 24 mm), head & neck (19 ± 40 mm), thorax 4D (18 ± 34 mm), and thorax 3D (21 ± 47 mm) remained within the lowest deviation range. Moderate overcoverage was seen for pelvis (39 ± 55 mm) and abdomen (77 ± 71 mm), while upper abdomen (120 ± 75 mm) and spine (155 ± 159 mm) exhibited the largest deviations, reflecting frequent scan extensions beyond SOP borders (see Fig. 1 for a visual representation of scan length deviations).

**Fig. 4:**
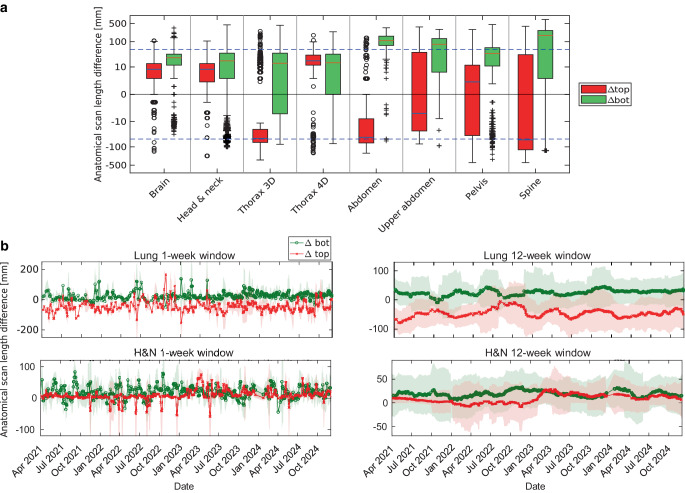
**a** Boxplots of anatomical scan length deviations from standard operating procedures (SOPs) at cranial (∆top, red) and caudal (∆bot, green) boundaries across body regions. Brain, head & neck (H&N), and 3D thorax show minimal deviations, while spine, abdomen, and upper abdomen exhibit larger ∆bot variability, indicating over-coverage. A symmetric log transformation improves visibility of small deviations and compresses outliers. Blue dashed lines indicate ±50 mm recommended thresholds [20]. **b** Temporal trends of anatomical scan length differences in ∆top and ∆bot for thorax 3D and head & neck protocols. Plots show 1‑week (left) and 12-week (right) moving averages; shaded areas represent standard deviation. Deviations remain stable with periodic fluctuations. 3D three-dimensional; 4D four-dimensional

S1 Table A3 in the supplementary information provides a detailed summary of anatomical scan length differences (∆top and ∆bot) across various protocols, showing mean and median deviations from SOP borders. Figure 4a illustrates both the distribution and temporal trends of these differences. In addition, in Fig. 4b weekly and 12-week intervals were analyzed to detect potential short-term variations related to rotating personnel, who are typically assigned to the CT for 1‑ to 2‑week periods. This analysis highlights notable variations across anatomical regions while confirming overall protocol consistency.

### Additional QA checks

An overview of the QA check results, including contrast agent application, metal artifact reduction, and topogram-to-volumetric scan length comparisons, is provided in Table 2. Representative values are provided here to highlight protocols with notable findings or high variability. For instance, contrast agent was applied in 16% of skull scans and 73% of head & neck scans, with volumes ranging from 40 to 120 ml across protocols, reflecting regional differences in contrast agent use. Metal artifacts were present in 93% of skull scans and 97% of H&N scans, but only a subset utilized iMAR for artifact reduction (39% and 93%, respectively), underscoring variability in metal management practices. Topogram-to-volumetric scan length analysis revealed that 10% of lung and 18% of pelvis scans extended beyond the topogram, indicating an opportunity for improvement.

**Table 2:** Summary of different quality assurance (QA) checks for different body regions. Contrast agent shows the percentage of cases with contrast applied and the contrast agent volume in milliliters, shown as the first to third quartile. Metal & iterative metal artifact reduction (iMAR) provides the percentage of scans containing metal and the percentage of scans using iMAR. Topogram-to-volumetric shows the frequency (in %) of cases where the topogram length was shorter than the volumetric scan length

	Skull	Head & neck	Breast	Lung	Upper abdomen	Abdomen	Pelvis	Spine
Contrast agent [%, ml]	16, 80	73, 88	12 , (70–120)	48, (40–120)	44, (65–120)	68, (85–120)	50, (50–120)	25, (65–120)
Metal & iMAR [%, %]	93, 39	97, 93	71, 21	79, 14	43, 8	34, 10	54, 11	72, 20
Topogram-to-volumetric [%]	2	5	11	10	6	18	18	9

Additionally, slice count compliance revealed five instances exceeding the 399-slice limit, with 4 cases in stereotactic brain scans and one in a head & neck scan. Breathing phase verification identified patients in an incorrect breathing phase in 56 breast irradiation scans (e.g. deep inspiration scans), 9 stereotactic lung scans (inspiration, expiration scans), and three stereotactic liver cases (inspiration, expiration scans). Bladder filling assessments showed that 93% of bladders in bladder scans were correctly empty, and 56% of all pelvic scans indicated an incorrectly empty bladder. In thorax-protocol-for-abdomen checks, 3% (27 cases) exhibited unexpected anatomical scan length difference, where the cranial border was underscanned (∆top < −100 mm), and the caudal border was overscanned (∆bot > 100 mm), likely due to the thorax protocol being applied to abdomen imaging. Lastly, FOV evaluations for axilla showed 72% scans with skin cuts (with one case false positive), while only 35% of the scans with cut skin used HDFoV.

## Discussion

We retrospectively analyzed imaging doses and scan lengths from over 13,300 radiotherapy CT scans acquired between April 2021 and December 2024, originating from 10,107 dose reports. The goal was to identify patterns in imaging dose metrics and anatomical coverage across protocols, assessing adherence to internal SOPs. Using TotalSegmentator, we systematically evaluated protocol compliance, revealing opportunities for quality improvement and dose optimization.

CT simulation is a cornerstone of radiotherapy, providing the anatomical and dosimetric foundation for treatment planning, verification and delivery. Its role in ensuring precise and safe treatment has been well established [42, 43]. High-quality, optimized imaging is thus essential for maintaining workflow integrity and patient safety.

Institutional CTDI_vol_ values and scan lengths generally aligned with reference levels from a UK-wide survey on radiotherapy CT protocols ([22]; Fig. 2). Higher imaging doses were observed in spine, head & neck, and brain protocols, likely due to a focus on image quality over dose reduction. For brain imaging, this is debatable, as MRI provides superior soft tissue contrast, and CT offers limited benefit. Dose-intensive manufacturer presets may have contributed and should be reassessed. In spine and head & neck protocols, tube modulation was initially based on “head” settings, resulting in unnecessarily high doses. Adjustments in 2024 to “neck” and “abdomen” modulation reduced dose by a factor of 3–10. Most scan lengths, except for brain and 4D protocols, exceeded the values proposed by Wood et al. [22], possibly due to prioritization of organ-at-risk capture over strict FOV limits. While CTDI_vol_ reflects scanner output rather than patient dose, it remains a useful standard for protocol comparison.

From a technical perspective, CT dose in head & neck and spine protocols was reduced by optimizing TCM to better match anatomical regions. CARE Dose 4D, which modulates tube current in real time based on patient size and region-specific needs [29, 30], offers five adaptation strengths to support this balance. Despite these capabilities, further dose reduction potential remains, especially for 4DCT, though many advanced methods are still in early stage research and not yet routine [44].

TotalSegmentator-based segmentation revealed that while overall protocol adherence was good, substantial scan length deviations existed in specific regions. Spine scans showed over-coverage and high variability (∆top: −68 ± 163 mm, Median (Med): −54 mm; ∆bot: 155 ± 159 mm, Med: 176 mm), and caudal overextension was common in upper abdomen (∆bot: 120 ± 75 mm, Med: 110 mm) and abdomen (∆bot: 77 ± 71 mm, Med: 80 mm). These findings point to opportunities for protocol refinement, particularly in spine and abdominal scans, where consistent field-of-view selection may be more challenging. As a potential improvement, aligning topogram acquisition with consistent breathing phases could help reduce variability in breathing-sensitive regions such as the thorax and abdomen.

While institutional CT protocols largely aligned with national guidelines, opportunities for further refinement remain. The German Society for Radiation Oncology [20] provides recommendations on FOV boundaries and tolerances to ensure consistency and accuracy. In radiotherapy, standardized treatment pathways support protocol adherence, and broader implementation of such guidelines could promote more uniform and reproducible imaging outcomes.

While SOPs guide radiotherapy CT protocols, clinical exceptions are often warranted to meet individual patient needs, potentially explaining some outliers. Moreover, an excessive number of scan protocols may confuse radiation therapy technologists (RTTs), especially in busy clinical environments where training levels vary. Standardized education and regular training could improve consistency and support protocol adherence. Beyond standardized training, regular feedback from the retrospective QA framework presented in this study could further enhance clinical practice if implemented in a live version. Such a system could provide near real-time insights into imaging dose and protocol adherence, either through cooperation with manufacturers for direct integration into scanner interfaces or through collaboration with medical physics experts (MPEs). Embedding this process into the daily routine of RTTs would enable continuous monitoring of planning CTs and facilitate proactive protocol optimization, thereby promoting sustainable QA across clinical workflows.

As CT technology advances, dose optimization remains critical for patient safety. Metal artifacts were common in skull (93%) and head & neck scans (97%), yet artifact reduction tools were inconsistently used, underscoring the need for standardized management. In response, decision trees were introduced to promote consistent use during protocol execution. Additionally, mismatches between topogram and scan lengths in 10% of lung and 18% of pelvis cases suggest suboptimal TCM, potentially affecting dose and image quality.

TotalSegmentator-based auto-segmentation tools are integral to radiotherapy workflows but require further validation to ensure consistent performance. Recent studies have demonstrated their clinical utility and robustness [40, 45, 46]. Incorporating intelligent dose management systems may enhance QA by enabling early detection of deviations or machine-related issues. While some reconstruction and artifact reduction tools in this study are vendor-specific, the proposed QA framework remains broadly applicable through vendor-neutral segmentation and analysis methods.

While optimizing CT protocols for dose reduction is important, it is crucial to recognize that the imaging dose is very small (~0.1%) compared to the therapeutic dose delivered during radiotherapy. Therefore, efforts to reduce imaging dose must be balanced with the need for accurate imaging to ensure effective treatment planning. In some cases, the potential benefit of dose reduction may be limited, and adjustments should be made with careful consideration of the overall radiation exposure in the context of the therapy [47]. Future work should also evaluate the impact of dose reduction on image quality and delineation accuracy, ideally through quantitative image analysis. Incorporating patient-specific parameters such as body mass index (BMI), height, or size-specific dose estimates (SSDE) could further personalize protocols and improve dose interpretation. In future implementations, reference lengths derived from the VB-LUT could be normalized to patient-specific anatomical dimensions, such as height or vertebral segment proportions, to further improve the interpretation of relative scan length deviations.

While this analysis was based on a single-institution cohort, the underlying methodology is broadly applicable and can be adapted to local PACS infrastructure and protocol definitions at other centers.

Moving forward, our institution is actively refining internal SOPs by fine-tuning the X‑ray tube output and defining optimized anatomical scan length boundaries, closely adhering to the latest guidelines. These measures aim to enhance protocol consistency and improve patient safety.

## Conclusion

This retrospective analysis showed that volumetric computed tomography dose index (CTDI_vol_) values were generally in line with expectations, though initial findings in spine, head & neck, and brain protocols indicated potential for dose optimization. Dose reductions have since been implemented in head & neck and spine imaging. Scan lengths were found to be generous overall, with greater anatomical variability in the abdomen and spine. These findings highlight opportunities to tighten scan boundaries in line with updated guidelines and reduce unnecessary radiation exposure.

## Supplementary Information

Overview. The Supplementary Information provides extended material supporting the quantitative and methodological analyses presented in the main manuscript. It includes: – Additional tables (Tables A1–A3) summarizing dose metrics, reference values from the literature, and detailed anatomical scan-length deviations across all CT body-region groups. – A detailed description of the vertebral-body lookup table (VB-LUT) used to estimate missing cranial and caudal coverage in truncated scans, together with an illustrative figure (Figure A1) demonstrating the application of the VB-LUT to a representative head-and-neck case. – An algorithmic overview of the full automated workflow for PACS retrieval, dose-report parsing, segmentation, and multi-step QA checks, provided as a schematic figure (Figure A2). This section documents the technical implementation of the retrieval pipeline, anatomical landmark extraction, and all integrated QA routines (contrast, metal, scan-range validation, breathing-phase verification, bladder filling, protocol misapplication, and FOV completeness).
