# Supplementary material for: Automated quality assurance of imaging dose and protocol adherence in computed tomography radiotherapy planning using TotalSegmentator-based segmentation
Source: Strahlenther Onkol. 2025 Nov 28;202(3):272–82. doi: 10.1007/s00066-025-02494-w (PMC12953283; doi:10.1007/s00066-025-02494-w)
Supplement: Supplementary file 1 — Overview. The Supplementary Information provides extended material supporting the quantitative and methodological analyses presented in the main manuscript. It includes: – Additional tables (Tables A1–A3) summarizing dose metrics, reference values from the literature, and detailed anatomical scan-length deviations across all CT body-region groups. – A detailed description of the vertebral-body lookup table (VB-LUT) used to estimate missing cranial and caudal coverage in truncated scans, together with an illustrative figure (Figure A1) demonstrating the application of the VB-LUT to a representative head-and-neck case. – An algorithmic overview of the full automated workflow for PACS retrieval, dose-report parsing, segmentation, and multi-step QA checks, provided as a schematic figure (Figure A2). This section documents the technical implementation of the retrieval pipeline, anatomical landmark extraction, and all integrated QA routines (contrast, metal, scan-range validation, breathing-phase verification, bladder filling, protocol misapplication, and FOV completeness). [file 66_2025_2494_MOESM1_ESM.docx]

# S1 - Supplementary Information – Extra Tables

**Table A1.** Summary of dose-related parameters for CT scans in external beam radiation therapy (EBRT) and brachyherapy. The table lists the number of patients (n) and the phantom diameter (PD, in cm) used to reference the equivalent CTDI in patient scans. Dose metrics are provided as mean ± standard deviation (SD) and median (Mdn) values, including CTDI_vol_ (mGy) as an indicator of radiation output and DLP (mGy·cm) representing cumulative dose over scan length (mm). Effective dose (E, in mSv) is estimated by applying the k-factor to DLP, based on AAPM standards [1]. Brachytherapy protocols are prefixed with “B”. Abbrevation: 4D – four-dimensional.

| **Examination** | **n** | **PD (cm)** | **CTDI_vol_  (mGy)** | | **DLP (mGy·cm)** | | **Scan length  (mm)** | | **k x 1000** | **E (mSv)** |
| --- | --- | --- | --- | --- | --- | --- | --- | --- | --- | --- |
|  |  |  | Mean ± SD | Mdn | Mean ± SD | Mdn | Mean ± SD | Mdn |  | Mean ± SD |
| 'Brain'  'Head & neck'  'Head & neck 24'  'B-Head & Neck'  'Axilla'  'Spine'  'Spine 24'  'Lung'  'Lung 4D'  'Breast'  'B-Breast'  'Upper Abdomen'  'Abdomen'  'Abdomen 4D'  'Prostate'  'B-Prostate'  'Cervix'  'B-Cervix'  'Pelvis'  'Legs' | 1441  1192  412  110  98  388  116  3382  843  1717  426  553  206  140  575  286  235  269  842  147 | 16  16  16  16  32  32  32  32  32  32  32  32  32  32  32  32  32  32  32  32 | 73 ± 12  56 ± 12  15 ± 1  37 ± 17  9 ± 7  60 ± 27  8 ± 3  7 ± 2  53 ± 20  8 ± 3  6 ± 2  8 ± 3  11 ± 4  55 ± 21  12 ± 3  10 ± 2  10 ± 4  10 ± 4  11 ± 4  9 ± 3 | 72  57  15  35  8  64  8  6  50  7  6  7  10  50  11  9  10  9  11  9 | 2066 ± 453  3212 ± 757  856 ± 121  621 ± 448  497 ± 458  3627 ± 1646  514 ± 231  320 ± 121  1641 ± 615  290 ± 114  179 ± 88  373 ± 178  612 ± 272  1623 ± 666  654 ± 223  167 ± 55  624 ± 284  371 ± 204  652 ± 247  713 ± 285 | 2011  3252  851  528  417  3866  478  305  1562  262  160  335  573  1467  626  157  583  306  618  695 | 287 ± 33  576 ± 58  579 ± 61  171 ± 45  542 ± 98  644 ± 168  646 ± 177  489 ± 62  319 ± 37  468 ± 47  235 ± 45  444 ± 82  566 ± 135  305 ± 49  561 ± 89  171 ± 36  594 ± 60  385 ± 119  600 ± 90  770 ± 141 | 283  576  575  166  527  612  625  486  319  465  231  440  585  319  574  168  590  344  601  749 | 2.1  3.1  3.1  3.1  3.1  3.1  3.1  14  14  14  14  15  15  15  15  15  15  15  15  15 | 4 ± 1  10 ± 2  3 ± 0  2 ± 1  2 ± 1  11 ± 5  2 ± 1  4 ± 2  23 ± 9  4 ± 2  3 ± 1  6 ± 3  9 ± 4  24 ± 10  10 ± 3  3 ± 1  9 ± 4  6 ± 3  10 ± 4  11 ± 4 |

**Table A2**. Achievable and proposed CTDI, scan length, and DLP values for different anatomical regions from Wood et al. [2].

| **Protocol** | **CTDI Achievable** (mGy) | **CTDI Proposed** (mGy) | **Scan Length Achievable** (mm) | **Scan Length  Proposed**  (mm) | **DLP Achievable** (mGy·cm) | **DLP Proposed** (mGy·cm) |
| --- | --- | --- | --- | --- | --- | --- |
| **Brain** | 42 | 50 | 250 | 290 | 1110 | 1500 |
| **Head & Neck** | 26 | 49 | 400 | 420 | 1080 | 2150 |
| **Lung 3D** | 10 | 14 | 370 | 390 | 410 | 550 |
| **Lung 4D** | 36 | 63 | 330 | 340 | 1170 | 1750 |
| **Breast** | 8 | 10 | 280 | 360 | 280 | 390 |
| **Cervix** | 12 | 16 | 380 | 400 | 510 | 610 |
| **Prostate** | 13 | 16 | 310 | 340 | 420 | 570 |

**Table A3.** Summary of anatomical scan length differences (Δtop and Δbot) for various CT bodyregion groups. Values represent the mean ± standard deviation (SD) and median anatomical scan length differences in mm from the anatomical scan lengths defined in the standard operating procedures (SOPs). Positive and negative differences indicate over-coverage and under-coverage, respectively, for each bodyregion group.

|  | | **Brain** (mm) | **H&N** (mm) | **Thorax 3D** (mm) | **Thorax 4D** (mm) | **Upper Abdomen** (mm) | **Abdomen** (mm) | **Pelvis** (mm) | **Spine** (mm) |
| --- | --- | --- | --- | --- | --- | --- | --- | --- | --- |
| **Δtop** | ***Mean*±*SD  Median*** | 11 ± 17 8 | 9 ± 32 8 | -45 ± 48 -47 | 17 ± 29 18 | -40 ± 51 -42.5 | 16 ± 65 -5 | -15 ± 57 2 | -68 ± 163 -54 |
| **Δbot** | ***Mean*±*SD Median*** | 24 ± 24 24 | 19 ± 40 18 | 21 ± 47 15 | 18 ± 34 15 | 120 ± 75 110 | 77 ± 71 80 | 39 ± 55 35 | 155 ± 159 176 |

# S2 - Supplementary Information – Vertebral Body Lookup Table Details

To standardize the estimation of negative scan length differences in truncated CT scans, a vertebral body lookup table (VB-LUT) was implemented. The VB-LUT defines representative anatomical reference lengths for the skull and vertebral body segments, based on typical adult anatomy.

| **Region** | **Anatomical Range** | **Reference Length (mm)** |
| --- | --- | --- |
| Skull | Entire skull | 210 |
| Cervical spine (C1–C2) | Atlas and axis | 25 |
| Cervical spine (C3–C7) | Lower cervical region | 20 |
| Thoracic spine (T1–T12) | Entire thoracic region | 25 |
| Lumbar spine (L1–L5) | Entire lumbar region | 30 |

For each scan, the vertebral levels within the reconstructed field of view were automatically identified from TotalSegmentator-derived segmentation [3]. If a scan was truncated at the cranial or caudal border, the missing length was estimated by summing the respective VB-LUT reference values for the absent vertebrae. Partially visible vertebral bodies were proportionally adjusted based on their visible fraction. Resulting deviations were recorded as Δtop (cranial) or Δbot (caudal), where positive values indicate overcoverage and negative values indicate undercoverage.


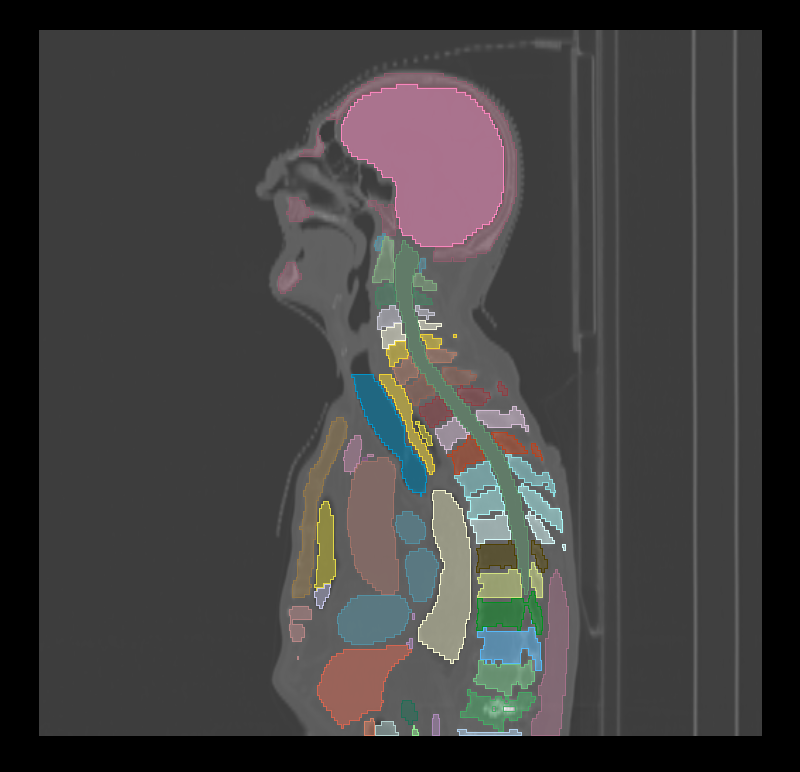

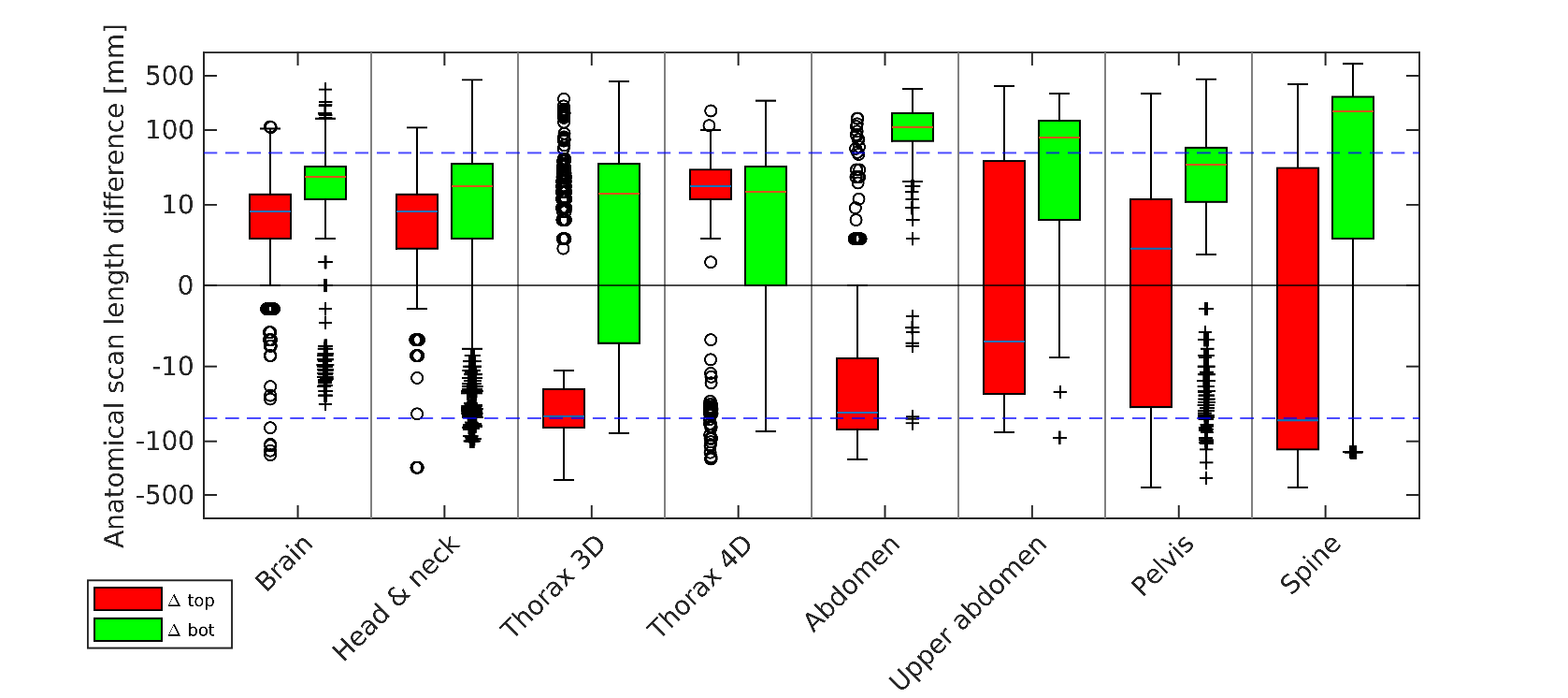


H&N


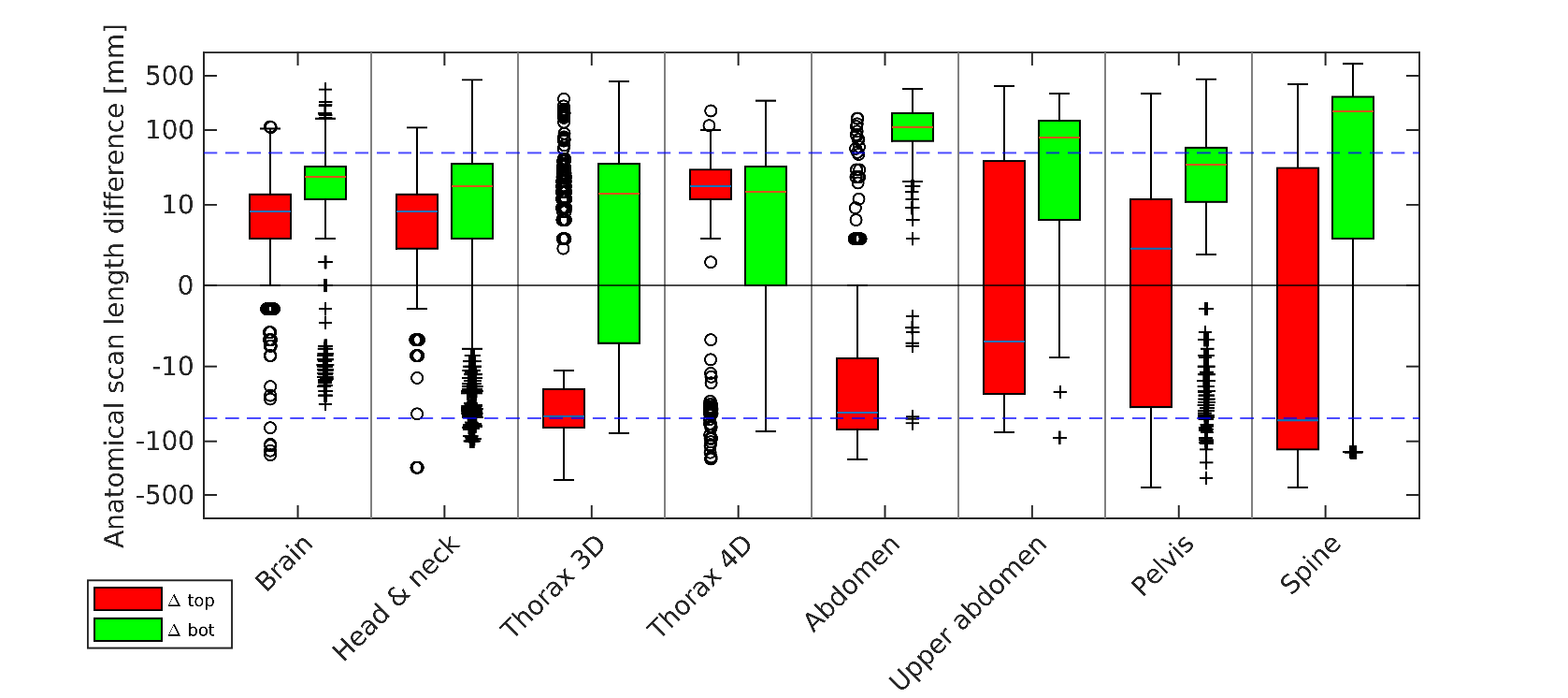

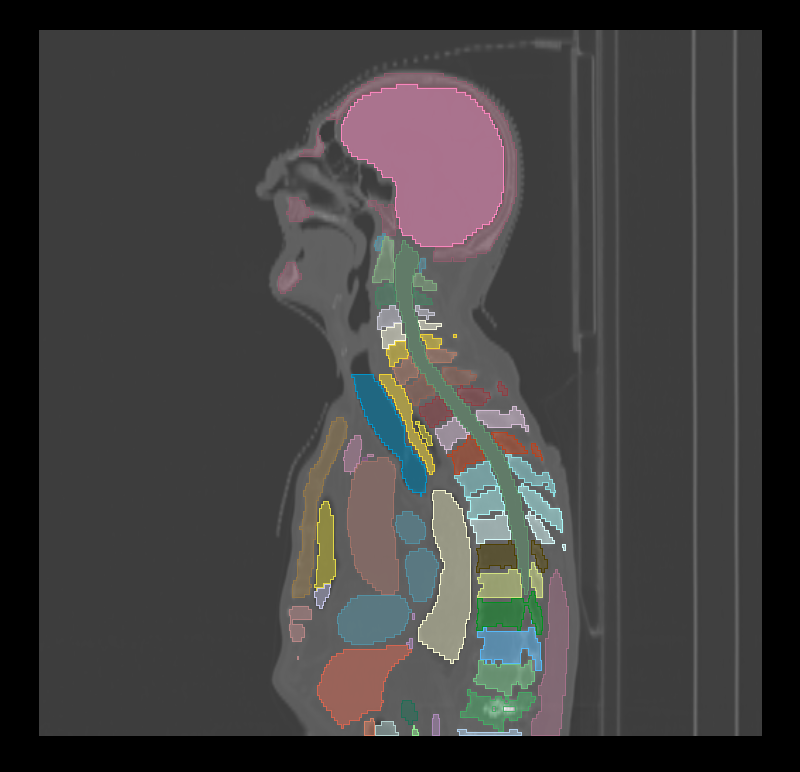


Missing vertebras

**Figure A1.** Illustration of the VB-LUT–based estimation of scan length deviations in a head & neck planning CT scan. Example of a planning CT after automated segmentation, demonstrating standardized quantification of anatomical coverage relative to SOP-defined scan borders. The red arrow (Δtop) indicates cranial overcoverage beyond the expected boundary, while the green arrow (Δbot) marks caudal truncation where parts of the lower thoracic vertebrae (T12, T11, and partially T10) are missing. Using the VB-LUT, the corresponding reference lengths of the missing vertebrae are summed to estimate the negative scan length difference. Segmentations were overlaid in 3D Slicer [4].

**S3 – Supplementary Information – Workflow and Algorithmic Overview**

This section summarizes the complete automated workflow for data retrieval, segmentation, and quality assurance (QA) of radiotherapy planning CT scans. The aim is to ensure reproducibility of the data processing pipeline while maintaining patient anonymity and institutional data protection requirements. An overview of the automated data retrieval, segmentation, and QA workflow is provided in Figure A2.

### Step 1 – Data Retrieval

All imaging studies were queried from the institutional Picture Archiving and Communication System (PACS) using the DCM4CHE [5] toolkit. A dedicated DICOM listener was configured within the local network to communicate directly with the institutional PACS, enabling both query and retrieve operations via standard DICOM C-FIND and C-MOVE services. A bash-based automation script managed the retrieval process for all radiotherapy-related CT series acquired between April 2021 and December 2024. Each retrieved dataset included:

- The imaging series (CT slices)
- The DICOM structured dose report
- The patient-level metadata (patient report)

Completeness of retrieved datasets was verified manually against the oncology information system (Mosaiq, Elekta AB, Stockholm, Sweden).

### Step 2 – Metadata Extraction and Aggregation

A MATLAB (version R2019b) script following Dave and Gingold [6] was developed to parse DICOM structured reports, extract dose and acquisition metadata (CTDIvol, DLP, scan length, and acquisition parameters), and convert them into a standardized tabular format (CSV). Key algorithmic components:

- Recursive parsing of DICOM tags across multi-event dose reports
- Aggregation of individual radiation events (e.g., inspiration/expiration in lung protocols) under a single dose report
- Error handling for incomplete or corrupted DICOM fields
- Validation through manual cross-checking of a subset of dose reports and metadata

### Step 3 – Segmentation and Anatomical Landmark Identification Planning CT images were automatically identified and segmented using the Python-based tool TotalSegmentator, which provides comprehensive organ and skeletal masks. These segmentation outputs enabled consistent anatomical referencing across patients and scan protocols.

The following anatomical landmarks were extracted for use in automated QA checks:

- Skull, cervical, thoracic, and lumbar vertebrae
- Diaphragm and lung borders
- Pelvic bones and femoral heads

These landmarks correspond to the anatomical field-of-view specifications summarized in Table 1 and were subsequently used to assign scans to anatomical regions and to estimate scan length deviations (see VB-LUT in Supplementary Section S2). Segmentations were visually reviewed in 3D Slicer only during the initial verification phase and for figure generation; the software was not part of the automated QA workflow.

### Step 4 – Additional Quality Assurance Checks

To ensure clinical protocol adherence and data integrity, a set of additional QA routines was implemented in MATLAB and integrated into the hierarchical QA decision framework. These checks complemented the dose- and segmentation-based evaluations described above.

Step 4.1 – Contrast and Metal Artifact Evaluation

Contrast agent usage was verified by extracting application volumes from the structured patient protocol report. The application of iterative metal artifact reduction (iMAR, Siemens Healthineers AG, Forchheim, Germany) consistency was assessed using voxel-based HU thresholding (HU > 4000) to detect metallic implants or prostheses and then check if the scan was reconstructed with iMAR.

Step 4.4.2 – Scan Range and Image Count Validation

Topogram and volumetric scan lengths were compared to ensure proper protocol coverage. Cases where the topogram length was shorter than the volumetric scan were marked as non-compliant. Additionally, image series containing fewer than 399 slices were verified as compliant with institutional limits, since the image guidance system at the treatment accelerator rejects scans exceeding this threshold.

Step 4.4.3 – Breathing Phase Verification (Thoracic Scans)

For thoracic acquisitions, segmented organ volumes—particularly lung segmentation—were used to verify the recorded breathing phase. When at least two scans with differing respiratory states (e.g., expiration and free breathing) were available, lung volumes were compared to confirm physiologically plausible differences between phases.

Step 4.4.4 – Bladder Filling Assessment (Pelvic Scans)

For pelvic imaging (bladder, rectal, prostate, anal, and cervical protocols), bladder volumes were automatically classified into three filling states: empty (<200 mL), partially filled (200–400 mL), and full (>400 mL). This check ensured adherence to the clinical recommendation for standardized bladder filling to minimize small bowel exposure.

Step 4.4.5 – Protocol Misapplication Detection

Protocol misapplication was evaluated by comparing the actual scan range with expected anatomical coverage. A thorax protocol was flagged as misapplied to an abdomen scan if the cranial boundary extended <-10 cm below and the caudal boundary extended >10 cm below the expected thoracic range, indicating incorrect protocol selection.

Step 4.4.6 – Field-of-View (FOV) Completeness Check (Axilla Protocols)

Axilla protocols were assessed for sufficient anatomical coverage. The reconstructed image was evaluated within a circular region corresponding to the scanner’s FOV. Non-air voxels (HU > –500) detected along the upper half of the FOV edge indicated potential truncation. Additionally, a predefined external check position was analyzed to identify if pixel values existed outside the standard reconstruction radius, which would signify the use of a High-Definition Field-of-View (HDFoV) reconstruction. Scans flagged by this automated check underwent manual review for confirmation, as complete anatomical coverage is clinically essential for these protocols.

**
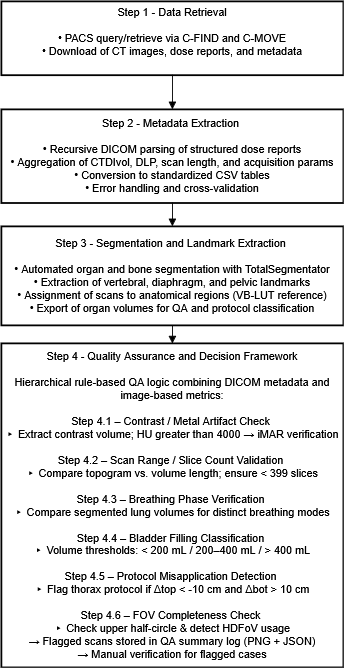
**

**Figure A2.** Algorithmic overview of the automated data-retrieval, segmentation, and QA workflow. The schematic summarizes the hierarchical processing pipeline for radiotherapy planning CT data. Step 1 covers PACS data retrieval using DCM4CHE and Bash automation, Step 2 structured report metadata extraction in MATLAB, Step 3 organ segmentation and landmark detection using TotalSegmentator, and Step 4 integrated MATLAB QA routines for contrast, metal, scan range, breathing phase, bladder filling, protocol misapplication, and FOV completeness.

**References**

[1] AAPM. *The Measurement, Reporting, and Management of Radiation Dose in CT - AAPM Task Group Report No. 96*. 2008; American Association of Physicists in Medicine: College Park, MD doi: <https://www.aapm.org/pubs/reports/RPT_96.pdf>

[2] Wood TJ, Davis AT, Earley J, Edyvean S, Findlay U, Lindsay R, et al. *IPEM topical report: the first UK survey of dose indices from radiotherapy treatment planning computed tomography scans for adult patients.* Phys Med Biol 2018;**63**:185008. doi: 10.1088/1361-6560/aacc87.

[3] Wasserthal J, Breit HC, Meyer MT, Pradella M, Hinck D, Sauter AW, et al. *TotalSegmentator: Robust Segmentation of 104 Anatomic Structures in CT Images.* Radiol Artif Intell 2023;**5**:e230024. doi: 10.1148/ryai.230024.

[4] Fedorov A, Beichel R, Kalpathy-Cramer J, Finet J, Fillion-Robin JC, Pujol S, et al. *3D Slicer as an image computing platform for the Quantitative Imaging Network.* Magn Reson Imaging 2012;**30**:1323-1341. doi: 10.1016/j.mri.2012.05.001.

[5] Warnock MJ, Toland C, Evans D, Wallace B, Nagy P. *Benefits of using the DCM4CHE DICOM archive.* J Digit Imaging 2007;**20 Suppl 1**:125-129. doi: 10.1007/s10278-007-9064-1.

[6] Dave JK, Gingold EL. *Extraction of CT dose information from DICOM metadata: automated Matlab-based approach.* AJR Am J Roentgenol 2013;**200**:142-145. doi: 10.2214/AJR.12.8501.
